# The anti-inflammatory effect of cyclooxygenase inhibitors in fibroblast-like synoviocytes from the human temporomandibular joint results from the suppression of PGE2 production

**DOI:** 10.1111/jop.12045

**Published:** 2013-01-18

**Authors:** Mutsumi Kawashima, Naomi Ogura, Miwa Akutsu, Ko Ito, Toshirou Kondoh

**Affiliations:** 1 Department of Maxillofacial Surgery, Nihon University School of Dentistry at MatsudoMatsudo, Chiba, Japan; 2 Research Institute of Oral Science, Nihon University School of Dentistry at MatsudoMatsudo, Chiba, Japan

**Keywords:** COX inhibitor, fibroblast-like synoviocyte, interleukin-6, prostaglandin E2, temporomandibular joint

## Abstract

**Background** Non-steroidal anti-inflammatory drugs (NSAIDs) have been widely used for the management of pain and inflammation. However, little remains known about the effects of NSAIDs on synovitis of the human temporomandibular joint (TMJ). The aims of this study were to investigate the potential anti-inflammatory effects of NSAIDs on synovitis of the TMJ and the inflammatory effects of PGE2 on fibroblast-like synoviocytes (FLS) derived from the TMJ.

**Methods** Human synovial tissue was obtained from patients with internal derangement who underwent arthroscopy of the TMJ. FLSs were prepared from the tissues using the outgrowth method. A COX inhibitor (indomethacin or celecoxib) was added to the IL-1β-stimulated cells in culture. The cells were also stimulated with PGE2 or an EP agonist. The PGE2 production and COX-2 and IL-6 expression levels were examined using enzyme-linked immunosorbent assays, real-time PCR, and a microarray analysis.

**Results** COX inhibitors decreased not only PGE2 production, but also the expression of COX-2 and IL-6 in FLS stimulated with IL-1β. EP2 and EP4 were both expressed in the FLS, and the treatment with EP2 and EP4 agonists induced IL-6 production in these cells.

**Conclusion** The COX inhibitors indomethacin and celecoxib reduce the expression of inflammatory factors, such as COX-2 and IL-6, in FLS from the TMJ via suppression of PGE2 production. EP2 and EP4 were the main receptors for PGE2 present in the FLS. The approach used in this study may be useful for revealing how drugs such as NSAIDs affect the cellular functions of FLS from the TMJ.

## Introduction

Synovitis, which often accompanies intracapsular pathological conditions, such as disk displacement (DD)/internal derangement (ID) and osteoarthritis (OA) of the temporomandibular joint (TMJ), is characterized by chronic inflammatory changes 1, 2. A number of mediators of inflammation (3–5) were detected in synovial fluid and tissue with intracapsular pathological conditions. Interleukin (IL)-1β, which is a pro-inflammatory cytokine that affects cell proliferation 6, inflammatory responses 7 and matrix remodeling 8, contributes to the progression of joint diseases such as rheumatoid arthritis (RA) 9. IL-1β has also been detected in the synovial fluid from patients with ID and/or OA of the TMJ 3–5. In a previous study performed to identify the putative genes associated with inflammation in synovitis of the TMJ, we used a microarray analysis to investigate the IL-1β-responsive genes in fibroblast-like synoviocytes (FLS) derived from patients with ID or OA 10.

Cyclooxygenase (COX)-2, which is also known as prostaglandin-endoperoxide synthase-2, was one of the major genes up-regulated in FLS stimulated with IL-1β 11. We also found the expression of prostaglandin (PG) E2 to increase in FLS stimulated with IL-1β 11. Previous reports have shown that the PGE2 level was increased in the synovial fluid from patients with ID and/or OA, or with RA 12–14. PGE2 also has been shown to modulate bone resorption by stimulating osteoclast formation from precursor stem cells 15, 16. All of these results suggest that PGE2 is implicated in the inflammation and tissue destruction that characterize arthritic diseases.

Non-steroidal anti-inflammatory drugs (NSAIDs) have been widely used for the management of pain and inflammation 17. The anti-inflammatory effects of NSAIDs are mainly due to their ability to inhibit COX, thus impairing the production of prostaglandins. COX enzymes metabolize arachidonic acid, forming PGH2, which is subsequently metabolized by prostaglandin E synthase into PGE2. Two isoforms of the COX enzyme exist 18. COX-1 is constitutively produced and functions in tissue homeostasis, whereas COX-2 is produced in response to stress, and is an inducible isoform that is up-regulated by a variety of cytokines and growth factors at sites of inflammation 19. Selective COX-2 inhibitors, such as celecoxib, are currently used for the suppression of PGE2 production because of their superior gastrointestinal safety compared with traditional non-selective NSAIDs 18, 20. Several reports have shown the effects of NSAIDs on TMJ inflammation induced by complete Freund’s adjuvant using model animals 21, 22. However, little remains known about the effects of NSAIDs on FLS, which play an important role in the pathological processes of synovitis in the TMJ.

The aim of this study was to investigate whether COX inhibitors controlled the inflammatory factors, including PGE2, on FLS of the TMJ. We examined the effects of COX inhibitors on the expression of inflammatory mediators in IL-1β-stimulated FLS. Indomethacin was used as a non-selective COX inhibitor and celecoxib was used as a COX-2 specific inhibitor for this study. We also examined the inflammatory effects of PGE2 and its receptor in FLS.

## Materials and methods

### Reagents

Recombinant human IL-1β was purchased from PeproTech, Inc. (Rocky Hill, NJ, USA). Ham’s F12 was obtained from Wako Pure Chemical Industries, Ltd. (Osaka, Japan). Foetal bovine serum (FBS) was obtained from Cell Culture Technologies, LLC (Gravesano, Switzerland). Penicillin G and kanamycin were purchased from Meiji Seika Kaisha, Ltd. (Tokyo, Japan), and fungizone was purchased from Chromogenix AB (Mölndal, Sweden). Indomethacin and PGE2 were purchased from Cayman Chemical Company (Ann Arbor, MI, USA), and celecoxib was purchased from Toronto Research Chemicals, Inc. (Toronto, ON, Canada). Selective EP agonists (ONO-DI-004 as an EP1 agonist, ONO-AE-259-1 as an EP2 agonist, ONO-AE-248 as an EP3 agonist, and ONO-AE1-329 as an EP4 agonist; each product was guaranteed to have >90% purity) were kind gifts from Ono Pharmaceutical Co., Ltd. (Osaka, Japan). The Affymetrix GeneChip® Human Genome Focus Array was obtained from Affymetrix (Santa Clara, CA, USA). The human IL-6 enzyme-linked immunosorbent assay (ELISA) kit was purchased from Thermo Scientific (Rockford, IL, USA), and the PGE2 kit was purchased from Invitrogen (Carlsbad, CA, USA).

### Cell culture

Human synovial tissue was obtained from patients with ID who underwent arthroscopy of the TMJ. The patients gave their complete informed consent for the surgery and for the use of their tissue in research studies. FLS samples from the TMJ synovium were prepared as previously described 23. The characteristics and symptoms of patients with ID are summarized in Table 1. The FLS were cultured with Ham’s F12 supplemented with 10% FBS, 100 μg/ml penicillin G, 100 μg/ml kanamycin, and 250 ng/ml fungizone. For the experiments, we used FLS from the sixth to eighth passages.

**Table 1:** Characteristics of patients with internal derangement

Patient No.	(Cell No.)	Sex	Age	Diseased side	ROM	Pain	Wilkes’s staging	Method
1	TMJI	F	23	L	+	+	II	Microarray analysis real lime-PCR ELISA
2	TMJ2	F	18	R	++	+	III	Microarray analysis
3	TMJ3	F	26	R	++	+	III	Microarray analysis
4	TMJ4	M	44	L	+	+	II	ELISA
5	TMJ5	M	M	R	++	+	II	Real-time PCR ELISA

M, male; F, female; R, right; L, left; ROM, range of motion; +, 30∼34mm; ++, 25∼29mm.

The experiments using FLS were performed according to the guidelines established by the Institutional Review Board of Nihon University School of Dentistry at Matsudo (Ethics Committee Registration Number: EC10-037).

### Enzyme-linked immunosorbent assay

Fibroblast-like synoviocytes were plated at 5 × 10^4^ cells per well in 24-well plates with Ham’s F12 medium supplemented with 10% FBS and antibiotics. Confluent cells were cultured for 24 h in media containing 2% FBS, and were then stimulated. For the experiments examining the effects of COX inhibitors, 1 μM or 10 μM indomethacin or 1 μM or 10 μM celecoxib were added to 100 pg/ml IL-1β-stimulated FLS cultures. To study the effects of PGE2 or the EP agonists, FLS were treated with 10 μM PGE2 or 10 μM of the EP agonist (ONO-DI-004 as an EP1 agonist, ONO-AE-259-1 as an EP2 agonist, ONO-AE-248 as an EP3 agonist, and ONO-AE1-329 as an EP4 agonist). The culture supernatants were then collected after the appropriate interval, and kept at −80°C until use. The PGE2 or IL-6 levels in conditioned media were measured using an ELISA kit.

### Total RNA extraction

The confluent-stage FLS were cultured in medium containing 2% FBS for 24 h, and then stimulated with or without 100 pg/ml IL-1β for various lengths of time. For the experiments examining the effects of COX inhibitors, 1 μM or 10 μM of indomethacin, or 1 μM or 10 μM of celecoxib, was added to the IL-1β-stimulated FLS cultures. The limit of detection of IL-1β is 100 pg/ml in the synovial fluid from the TMJ of patients with OA, based on previous reports 5–23. Total cellular RNA from FLS was extracted using the miRNeasy Mini Kit, (Qiagen, Valencia, CA, USA), and then stored at −80°C until use.

### Real-time polymerase chain reaction (PCR)

Complementary DNA was synthesized using a GeneAmp® RNA PCR kit (Perkin-Elmer Corporation, Norwalk, CT, USA). Real-time PCR was performed with a DyNAmo SYBR Green qPCR kit (Finnzymes, Espoo, Finland). The PCR mixture, containing 20 pmol of forward and reverse primers and 2 μl of cDNA, was subjected to amplification with a DNA Engine Opticon® 1 (Bio-Rad, Hercules, CA, USA), with pre-heating at 95°C for 10 min, followed by 40 cycles of 94°C for 15 s, 55°C for 30 s, and 72°C for 30 s. Amplicons were directly detected by measuring the increase in fluorescence caused by the binding of the SYBR Green I dye to gene-specific, amplified, double-stranded DNA, using a DNA Engine Opticon® 1. After the PCR was complete, the temperature was raised from the annealing temperature to 95°C for a melting curve analysis. The primer sequences used for the real-time PCR analysis are shown in Table 2.

**Table 2:** Primers for genes

Genes	Primer	Products size (bp)
COX-2	F:5′-TTC AAA TGA GAT TGT GGG AAA ATT GCT-3′	304
	R:5′-AGA TCA TCT CTG CCT GAG TAT CTT-3′	
IL-6	F:5′-CCA CTC ACC TCT TCA GAA-3′	453
	R:5′-GCG CAA AAT GAG ATG AGT-3′	
EP2	F:5′-GCT ATC ATG ACC ATC ACC TT-3′	109
	R:5′-ACC TAA GAG CTT GGA GGT C-3′	
EP4	F:5′-CAT CTT ACT CAT TGC CAC CT-3	112
	R:5′-TTT ACT GAC TTC TCG CTC CA-3′	
GAPDH	F:5′-ATC ACC ATC TTC CAG GAG-3′	315
	R:5′-ATG GAC GTG GGT CAT GAG-3′	

COX-2, cyclooxygenase-2; IL-6, interleukin-6; EP, E-prostanoid receptor; GAPDH, glyceraldehydes-3-phosphate dehydrogenase.

The initial template concentration was derived from the cycle number at which the fluorescent signal crossed a threshold in the exponential phase of the PCR (CT-value). The number of transcripts was determined based on the threshold cycle of each experimental gene and of glyceraldehyde-3-phosphate dehydrogenase (GAPDH). The relative abundance of the gene transcripts vs. GAPDH was indicated by ΔCT (CT-experimental gene minus CT-GAPDH). The ΔΔCT (ΔCT-treated minus ΔCT-none) indicates the relative *n*-value of the expression of each gene compared with the same gene expression in the control. The value 2^−*n*^ indicates the relative expression of experimental genes as the fold change vs. the expression level in an untreated sample. All analyses were performed in triplicate, and the results were confirmed by three independent experiments.

### Microarray analysis

For gene expression profiling, we used the Affymetrix GeneChip® Human Genome Focus Array according to Affymetrix protocols. Raw data from 10 GeneChips were loaded into the GeneSpring GX software program (Agilent Technologies, Santa Clara, CA, USA). Data were normalized using the median raw data from each array as a reference. The changes in gene expression were determined by comparing the average normalized intensities for untreated cells with those of IL-1β-treated cells.

### Statistical analysis

The data were expressed as the means ± standard deviations and were analyzed using a one-way analysis of variance (ANOVA).

## Results

### Effects of COX inhibitors on PGE2 generation

**Figure 1:**
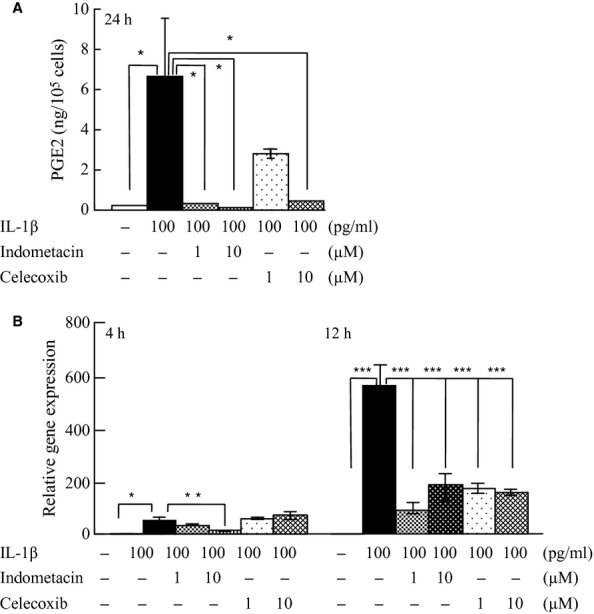
Effect of COX inhibitors on PGE2 production and COX-2 expression. (A) The levels of PGE2 production in the conditioned media from fibroblast-like synoviocytes (FLS) were determined using an ELISA. The cells were cultured with or without IL-1β and COX inhibitors, and incubated for 24 h. (B) The levels of COX-2 gene expression in the FLS were examined using real-time PCR. The cells were cultured with or without IL-1β and COX inhibitors, and incubated for 4 and 12 h. *n* = 4, *P < 0.05, **P < 0.01, ***P < 0.005.

To examine the effect of COX inhibitors on PGE2 generation, FLS were treated with 1 μM or 10 μM indomethacin or 1 μM or 10 μM celecoxib after being stimulated with IL-1β. The production of PGE2 was significantly increased by 100 pg/ml IL-1β in the FLS, and was significantly decreased by exposure to 1 μM or 10 μM indomethacin and 10 μM celecoxib for 24 h (Fig. 1A). The gene expression of COX-2 was also significantly increased by IL-1β in the FLS exposed to the inhibitors for both 4 and 12 h, and was significantly decreased following a 4-h exposure to 10 μM indomethacin or a 12-h exposure to 1 μM or 10 μM of either indomethacin or celecoxib (Fig. 1B).

### Effect of COX inhibitors on IL-6 expression

**Figure 2:**
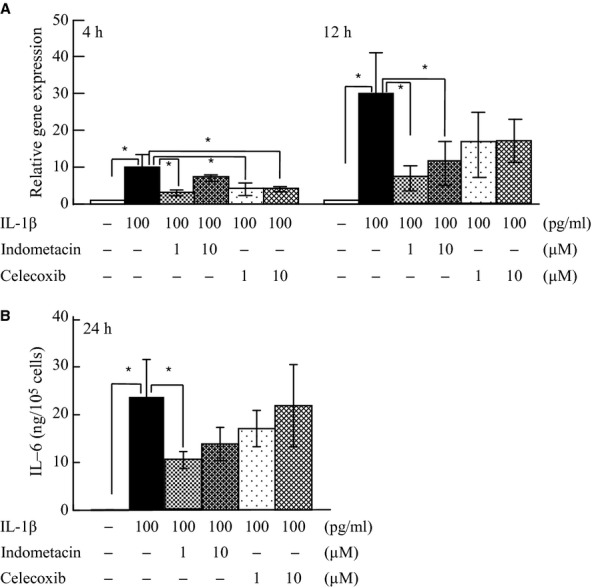
Effect of COX inhibitors on IL-6. (A) The levels of IL-6 gene expression in fibroblast-like synoviocytes (FLS) were determined by real-time PCR. The cells were cultured with or without IL-1β and COX inhibitors, and incubated for 4 and 12 h. (B) The levels of IL-6 protein production in the conditioned medium from FLS was determined by an ELISA. The cells were cultured with or without IL-1β and COX inhibitors, and incubated for 24 h. *n* = 4, *P < 0.05, **P < 0.01, ***P < 0.005.

To examine the anti-inflammatory effect of COX inhibitors, the gene expression and protein production of IL-6 were measured in IL-1β-stimulated FLS treated with or without COX inhibitors. As shown by the microarray analysis in our previous report 10, IL-6, which has an important role in the pathology of inflamed joints, such as in RA 24, was significantly up-regulated in FLS stimulated by IL-1β. The 1 μM concentration of indomethacin significantly reduced both the gene and protein expression of IL-6 in the FLS stimulated with IL-1β at all time points examined (Figs. 2A,B). The IL-6 production was found to be significantly increased in FLS stimulated with IL-1β for 24 h (Fig. 2B) In contrast, celecoxib only slightly decreased the gene and protein expression of IL-6 in IL-1β-stimulated FLS, and this difference was not significant compared with FLS incubated with only IL-1β (Figs. 2A,B).

### EP expression in FLS

**Figure 3:**
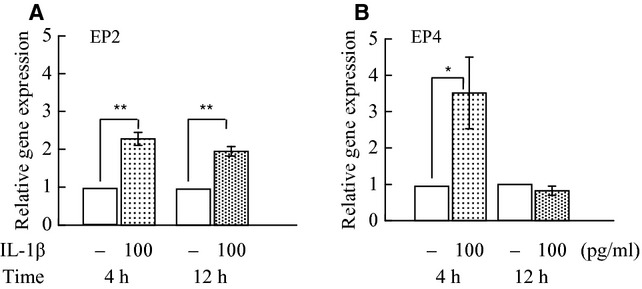
The gene expression levels of EP2 and EP4 in fibroblast-like synoviocytes were determined using real-time PCR. (A) EP2 gene expression. (B) EP4 gene expression. The cells were cultured with or without IL-1β, and were incubated for 4 or 12 h. *n* = 4, *P < 0.01. **P < 0.001.

COX inhibitors reduce prostaglandin generation by inhibiting the enzymatic activities of COX-1 and/or COX-2. As shown in Figs 1 and 2, the administration of COX inhibitors decreased the gene expression of COX-2 and IL-6. Therefore, we hypothesized that the reduced expression of COX-2 and IL-6 may occur concomitantly with a decrease in PGE2 production in IL-1β-stimulated FLS treated with COX inhibitors. PGE2 induces its effects through binding to four specific cell-surface receptors, the E-prostanoid (EP) receptors (EP1 to EP4) 15,24. We examined the expression levels of the four EP subtypes, EP1, EP2, EP3, and EP4, in FLS. Table 3 summarizes the data regarding expression of the EP receptors in FLS, as determined by a microarray analysis. The expression of the EP2 and EP4 genes was detected in FLS, whereas the expression of EP1 and EP3 was not detected. Real-time PCR confirmed the expression of EP2 and EP4 in FLS regardless of whether they were stimulated with IL-1β. The EP2 expression was enhanced in the FLS stimulated with IL-1β for both 4 h and 12 h (Fig. 3A). In contrast, the EP4 expression was enhanced in FLS stimulated with IL-1β for 4 h, and then was decreased in FLS stimulated with IL-1β for 12 h (Fig. 3B).

**Table:** Expression of EP receptor (EP1-4) genes in FLS by microarray

	EP1	EP2	EP3	EP4
Intensity	Control	A	2.802 ± 1.215	A	0.620 ± 0.267
	IL-1β	A	6.762 ± 3.078	A	1.519 ± 0.568
Fold	IL-1β/control	(–)	2.653 ± 1.064	(–)	2.763 ± 1.379

*N* = 5; A, absent; Fold, average normalized intensity of IL-1β-stimulated FLS/average normalized intensity of control FLS.

### Effects of specific EP agonists on IL-6 production

**Figure 4:**
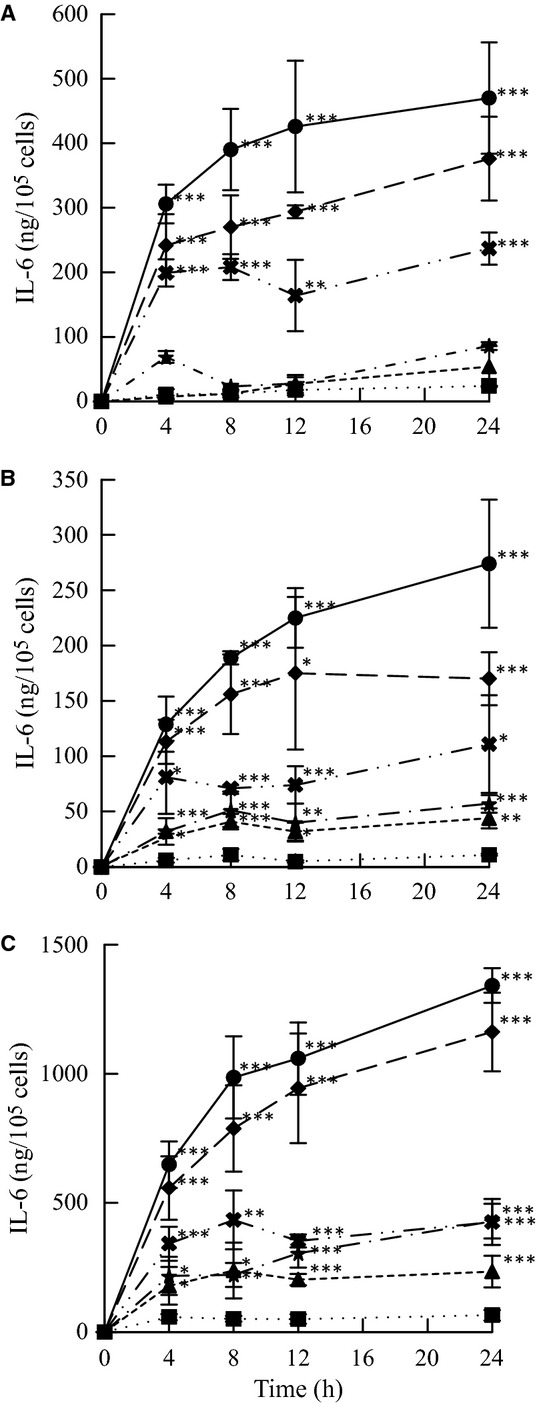
Effect of EP agonists on IL-6 production. A–C show the results of different samples. The time course of IL-6 protein production in the conditioned medium of FLS was determined by an ELISA. The cells were cultured with or without PGE2 or EP receptor agonists, and were incubated for 4, 8, 12, or 24 h. 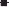 None, 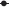 PGE2, 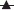 EP1 agonist (ONO-DI-004), 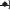 EP2 agonist (ONO-AE-259-1), 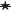 EP3 agonist (ONO-AE-248), 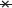 EP4 agonist (ONO-AE1-329), *P < 0.05, **P < 0.001, ***P < 0.005.

As the expression of EP receptors was detected in FLS, we examined the effect of PGE2 on IL-6 production in the FLS from three patients with ID. In all three FLS samples from the three patients, the IL-6 production was increased in the cells stimulated by PGE2 in a time-dependent manner (Fig. 4). We also examined the effect of specific EP1 to EP4 agonists on the IL-6 production of the FLS. Among the EP agonists, the EP2 agonist, ONO-AE-259-1, most effectively stimulated the IL-6 production in FLS. The effects on the levels of IL-6 occurred in the order: PGE2 > ONO-AE-259-1 (EP2 agonist) > ONO-AE1-329 (EP4 agonist) > ONO-AE-248 (EP3 agonist) > ONO-DI-004 (EP1 agonist) in all three FLS samples, although the levels of IL-6 production were different in each patient (Fig. 4). The EP1 agonist (ONO-DI-004) and the EP3 agonist (ONO-AE-248) increased the IL-6 production in FLS from Patient #2 and Patient #3, whereas these agonists did not affect the IL-6 production in FLS from Patient #1.

## Discussion

This study demonstrated that COX inhibitors decreased not only the PGE2 production but also the expression of COX-2 and IL-6, in FLS stimulated with IL-1β (Figs 1 and 2), which is a strong inducer of PGE2 and IL-6 in FLS. The suppression of IL-1β-induced PGE2 secretion by celecoxib was dose-dependent (Fig. 1A). However, both concentrations of celecoxib suppressed COX-2 mRNA production equally (Fig. 1B). These results suggest that the effect resulting from exposure to 1 μM celecoxib diminishes earlier than that associated with exposure to indomethacin or 10 μM celecoxib.

Previous reports have shown that the levels of both PGE2 and IL-6 were increased in the synovial fluid of patients with ID and/or OA in the TMJ 4, 12, 13, 26. IL-6, which has important roles in immune responses and bone resorption, is one of the pathological factors involved in not only RA but also osseous changes in the TMJ 27–28. These results suggest that COX inhibitors are useful for the suppression of inflammation and bone destruction in the TMJ.

The COX inhibitors, which are known to reduce prostaglandin generation by inhibiting COX enzyme activity 21, down-regulated the gene expression of COX-2 and IL-6 in this study (Figs 1 and 2). The observed reduction in COX-2 and IL-6 expression may reflect, at least partially, a decrease in the autocrine effect of PGE2 resulting from exposure to the COX inhibitors. PGE2 has been shown to be a principal mediator of inflammation in diseases such as RA and OA 13. It has also been reported that the level of PGE2 was increased in inflammatory TMJ models in experimental animals 11. However, little remains known about the effect of PGE2 in inflammation of the TMJ in humans. Therefore, we examined whether PGE2 affects the inflammatory responses in FLS.

PGE2 exerts its effects through a family of G protein-coupled receptors: EP1, EP2, EP3, and EP4 29. We examined whether these receptors mediated the biological function of PGE2 in FLS using specific EP agonists. The EP2 agonist, ONO-AE-259-1, had the strongest stimulatory effect on IL-6 production in the FLS (Fig. 4). Therefore, the IL-6 production in FLS by PGE2 was mainly mediated by the EP2 receptor, which is in agreement with the fact that it had the highest level of expression among the EP receptors in FLS, as shown by a microarray analysis (Table 2). In addition, EP2 was continuously enhanced in FLS stimulated with IL-1β, as seen by real-time PCR (Fig. 3). On the other hand, the EP4 receptor was partially effective with regard to the IL-6 production in FLS mediated by PGE2 as indicated by treatment with the EP4 agonist, ONO-AE1-329. The expression of the EP4 receptor was at a lower level than that of the EP2 receptor, and was transiently enhanced by IL-1β (Fig. 3). In contrast, the effects of the EP1 and EP3 receptors on IL-6 production were small in FLS stimulated with PGE2. The EP1 agonist (ONO-DI-004) and the EP3 agonist (ONO-AE-248) increased the IL-6 production slightly in FLS samples from two patients, and produced no effect in the FLS sample from one patient (Fig. 4). Our microarray analysis did not detect expression of EP1 and EP3 in FLS (Table 2). Although the expression levels of EP1 and EP3 were different in individual TMJ patients, these receptors had little or no impact on the expression of IL-6.

It has been reported that FLS from the knees of patients with OA and RA expressed EP2, EP3, and EP4 mRNAs, but not EP1 mRNA, although there were differences in the expression of the EP3 receptor among the RA donors. In addition, the expression of mRNAs encoding EP2 and EP4 was up-regulated by IL-1β treatment in FLS from patients with OA and RA 30. The up-regulation of the EP2 and EP4 receptors has also been reported in the synovial tissue of rats with adjuvant arthritis 31. Furthermore, previous reports have shown that PGE2 stimulated the production of IL-6 in FLS from patients with OA and/or RA through the EP2 and/or EP4 receptors 30. It has been reported that EP2 and EP4 receptors, rather than EP3 receptors, are also abundantly expressed in human articular cartilage, thus suggesting that the PGE2/EP2 and/or PGE2/EP4 signaling pathway may be clinically involved in the onset and progression of OA 32. Based on these results, PGE2 signaling through EP2/EP4 may be a key part of not only synovitis but also of cartilage inflammation of various joints.

We examined the biological effect of two COX inhibitors, indomethacin and celecoxib, in the FLS from patients with inflammatory conditions. Indomethacin significantly reduced the elevated PGE2 and IL-6 production in FLS that had been stimulated with IL-1β. In contrast, celecoxib slightly decreased the gene expression and the protein production of IL-6 in IL-1β-stimulated FLS. COX-1 was constitutively expressed in the FLS from patients with TMJ 11. Therefore, the PGE2 production was reduced more in the FLS of patients who had received indomethacin than in the FLS of patients who had received celecoxib. In addition, indomethacin also reduced the expression of COX-2 and IL-6 more strongly than did celecoxib, reflecting the decreased level of PGE2 production. In contrast to these findings, recent studies have reported that the anti-inflammatory effect of COX inhibitors may not occur exclusively through their inhibition of COXs, but rather, may occur as a consequence of the effects of these drugs via many different pathways, including the direct inhibition of nuclear factor (NF)-κB 33. Further studies are needed to elucidate the exact mechanism of action of COX inhibitors, as they might be of great biological and therapeutic significance in synovitis, ID, and OA of the TMJ 34.

Currently, conservative approaches, such as splinting and physical therapy, are the main treatments for ID. We recently performed a few surgical procedures to treat ID of the TMJ. Since the TMJ is a small joint space compared with other joints (e.g., the shoulder, knee, and hip), this study was limited by the difficulty of obtaining a sufficient quantity of synovial fibroblasts. We therefore performed only a few surgical procedures for ID/OA of the TMJ. In addition, ethical concerns prohibited the collection of healthy synovia. We therefore isolated FLS from portions of synovial tissues from patients experiencing inflammation and other symptoms of joint disease, and the FLS were then stimulated with IL-1β in an *in vitro* condition simulating synovitis. Further studies should be conducted to compare FLS from synovial tissues of patients with different joint disease conditions and/or different Wilkes’s stages.

**Figure 5:**
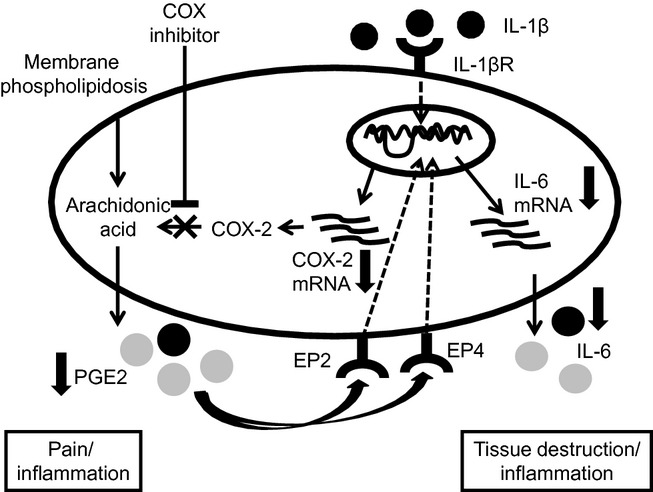
Effect of COX inhibitors on the levels of PGE2 and IL-6.

In conclusion, the results of this study indicate that one of the anti-inflammatory effects of the COX inhibitors indomethacin and celecoxib is to reduce the gene expression of COX-2 and IL-6 in FLS from the TMJ, as shown in Fig. 5. Additionally, PGE2 affects IL-6 production through EP2 and EP4 in the FLS. Our results suggest that these COX inhibitors are useful for treating synovitis in TMJ through the suppression of not only PGE2 but also the inflammatory mediators such as IL-6.

## References

[1] Carls FR, von Hochstetter A, Makek M, Engelke W (1995). Diagnostic accuracy of TMJ arthroscopy in correlation to histological findings. J Craniomaxillofac Surg.

[2] Dijkgraaf LC, Liem RSB, de Bont LGM (1997). Synovial membrane involvement in osteoarthritic temporomandibular joints. A light microscopic study. Oral Surg Oral Med Oral Pathol Oral Radiol Endod.

[3] Murakami K, Segami N, Fujimura K, Iizuka T (1991). Correlation between pain and synovitis in patients with internal derangement of the temporomandibular joint. J Oral Maxillofac Surg.

[4] Kubota E, Kubota T, Matsumoto J, Shibata T, Murakami K (1998). Synovial fluid cytokines and proteinases as markers of temporomandibular joint disease. J Oral Maxillofac Surg.

[5] Takahashi T, Kondoh T, Fukuda M, Yamazaki Y, Toyosaki T, Suzuki R (1998). Proinflammatory cytokines detectable in synovial fluids from patients with temporomandibular disorders. Oral Surg Oral Med Oral Pathol Oral Radiol Endod.

[6] Bucala R, Ritchlin C, Winchester R, Cerami A (1991). Constitutive production of inflammatory and mitogenic cytokines by rheumatoid synovial fibroblasts. J Exp Med.

[7] Dinarello CA, Ikejima T, Warner SJC (1987). Interleukin 1 induces interleukin 1. I. Induction of circulating interleukin-1 in rabbits in vivo and in human mononuclear cells in vitro. J Immunol.

[8] Vincenti MP, Brinckerhoff CE (2001). Early response genes induced in chondrocytes stimulated with the inflammatory cytokine interleukin-1β. Arthritis Res.

[9] Sweeney SE, Firestein GS (2004). Rheumatoid arthritis: regulation of synovial inflammation. Int J Biochem Cell Biol.

[10] Ogura N, Akutsu M, Tobe M, Sakamaki H, Abiko Y, Kondoh T (2007). Microarray analysis of IL-1β-stimulated chemokine genes in synovial fibroblasts from human TMJ. J Oral Pathol Med.

[11] Satoh K, Ogura N, Akutsu M (2009). Expression of cyclooxygenase-1 and -2 in IL-1β-induced synovitis of temporomandibular joint. J Oral Pathol Med.

[12] Arinci A, Ademoglu E, Aslan A, Mutlu-Turkogle U, Karabulut AB, Karan A (2005). Molecular correlates of temporomandibular joint disease. Oral Surg Oral Med Oral Pathol Oral Radiol Endod.

[13] Park JY, Pillinger MH, Abramson SB (2006). Prostaglandin E2 synthesis and secretion: the role of PGE2 synthases. Clinical Immunology.

[14] Lee YA, Choi HM, Lee SH (2012). Synergy between adiponectin and interleukin-1β on the expression of interleukin-6, interleukin-8, and cyclooxygenase-2 in fibroblast-like synoviocytes. Exp Mol Med.

[15] Martel-Pelletier J, Pelletier JP, Fahmi H (2003). Cyclooxygenase-2 and prosta-glandins in articular tissues. Semin Arthritis Rheum.

[16] Zuo W, Wu Z, Wu N (2011). Adiponectin receptor 1 mediates the difference in adiponectin-induced prostaglandin E2 production in rheumatoid arthritis and osteoarthritis synovial fibroblasts. Chin Med J(Engl).

[17] Paiotti AP, Marchi P, Miszputen SJ, Oshima CT, Franco M, Ribeiro DA (2012). The role of nonsteroidal antiinflammatory drugs and cyclooxygenase-2 inhibitors on experimental colitis. In Vivo.

[18] Zweers MC, de Boer TN, van Roon J, Bijlsma JWJ, Lafeber FPJG, Mastbergen SC (2011). Celecoxib: considerations regarding its potential disease-modifying properties in osteoarthritis. Arthritis Res Ther.

[19] Taketa T, Sakai A, Tanaka S (2008). Selective cyclooxygenase-2 inhibitor prevents reduction of trabecular bone mass in collagen-induced arthritic mice in association with suppression of RANKL/OPG ratio and IL-6 mRNA expression in synovial tissues but not in bone marrow cells. J Bone Miner Metad.

[20] McCormack PL (2011). Celecoxib: a review of its use for symptomatic relief in the treatment of osteoarthritis, rheumatoid arthritis and ankylosing spondylitis. Drugs.

[21] Schutz TCB, Andersen ML, Tufik S (2007). Effects of COX-2 inhibitor in temporomandibular joint acute inflammation. J Dent Res.

[22] Kerins C, Carlson D, Mclntosh J, Bellinger L (2004). A role for cyclooxygenase II inhibitors in modulating temporomandibular joint inflammation from a meal pattern analysis perspective. J Oral Maxillofac Surg.

[23] Ogura N, Tobe M, Sakamaki H (2002). Interleukin-1β induces interleukin-6 mRNA expression and protein production in synovial cells from human temporomandibular joint. J Oral Pathol Med.

[24] Fonseca JE, Santos MJ, Canhao H, Choy E (2009). Interleukin-6 as a key player in systemic inflammation and joint destruction. Autoimmun Rev.

[25] Kunisch E, Jansen A, Kojima F (2009). Prostaglandin E2 differentially modulates proinflammatory/prodestructive effects of TNF-α on synovial fibroblasts via specific E prostanoid receptors/cAMP. J Immunol.

[26] Kaneyama K, Segami N, Sato J, Nishimura M, Yoshimura H (2004). Interleukin-6 family of cytokines as biochemical markers of osseous changes in the temporomandibular joint disorders. Br J Oral Maxillofac Surg.

[27] Le Goff B, Blanchard F, Berthelot JM, Heymann D, Maugars Y (2010). Role of interleukin-6 in structural joint damage and systemic bone loss in rheumatoid arthritis. Joint Bone Spine.

[28] Palmqvist P, Persson E, Conaway HH, Lernaer UH (2002). IL-6, leukemia inhibitory factor, and oncostatin M stimulate bone resorption and regulate the expression of receptor activator of NF-kB ligand, osteoprotegerin, and receptor activator of NF-kB in mouse calvariae. J Immunol.

[29] Kojima F, Naraba H, Sasaki Y, Beppu M, Aoki H, Kawai S (2003). Prostaglandin E2 is an enhancer of interleukin-1β-induced expression of membrane-associated prostaglandin E synthase in rheumatoid synovial fibroblasts. Arthritis Rheum.

[30] Inoue H, Takamori M, Shimoyama Y, Ishibashi H, Yamamoto S, Koshihara Y (2002). Regulation by PGE2 of the production of interleukin-6, macrophage colony stimulating factor, and vascular endothelial growth factor in human synovial fibroblasts. Br J Pharmacol.

[31] Kurihara Y, Endo H, Akahoshi T, Kondo H (2001). Up-regulation of prostaglandin E receptor EP2 and EP4 subtypes in rat synovial tissues with adjuvant arthritis. Clin Exp Immunol.

[32] Li X, Ellman M, Muddasani P (2009). Prostaglandin E2 and its cognate EP receptors control human adult articular cartilage homeostasis and are linked to the pathophysiology of osteoarthritis. Arthritis Rheum.

[33] Park JH, Han DC, Kim J (2006). Differential regulation of anti-inflammatory proteins in human rheumatoid synoviocytes MH7A cell by celecoxib and ibuprofen. Life Sci.

[34] Yang SF, Hsieh YS, Lue KH, Chu SC, Chang IC, Lu KH (2008). Effects of nonsteroidal anti-inflammatory drugs on the expression of urokinase plasminogen activator and inhibitor and gelatinases in the early osteoarthritic knee of humans. Clin Biochem.

